# Autopsy Findings in Case of Fatal Scorpion Sting: A Systematic Review of the Literature

**DOI:** 10.3390/healthcare8030325

**Published:** 2020-09-06

**Authors:** Alessandro Feola, Marco Alfonso Perrone, Amalia Piscopo, Filomena Casella, Bruno Della Pietra, Giulio Di Mizio

**Affiliations:** 1 Department Experimental Medicine, University of Campania, 80138 Naples, Italy; bruno.dellapietra@unicampania.it; 2 Division of Cardiology, University of Rome “Tor Vergata”, 00133 Rome, Italy; marco.perrone01@gmail.com; 3 Forensic Medicine, Department of Law, “Magna Graecia” University of Catanzaro, 88100 Catanzaro, Italy; amaliapiscopo@gmail.com (A.P.); giulio.dimizio@unicz.it (G.D.M.); 4 Unit of Legal Medicine, AORN “Sant’Anna e San Sebastiano”, 81100 Caserta, Italy; mena.casella84@gmail.com

**Keywords:** autopsy, scorpion, toxicology, ARDS, pulmonary edema

## Abstract

Scorpion sting is a public health issue in several countries, particularly in America, the Middle East, India and Africa. The estimated annual global incidence of scorpion envenomings is about 1.5 million, resulting in 2600 deaths. Scorpions are Arthropoda characterized by a tail ending in a terminal bulbous (telson) containing paired venom glands and the stinger. There are 19 known families of scorpions and more than 2200 species, of which about 50 from the families of Buthidae, Hemiscorpiidae and Scorpionidae are harmful to humans. Scorpion venom is a complex structure composed of neurotoxic proteins, salts, acidic proteins and organic compounds, thereby having neurologic, cardiovascular, hematologic and renal side effects, in addition to local effects such as redness, pain, burning and swelling. When the sting is fatal, the mechanism of death is often related to cardiotoxicity with terminal pulmonary edema. However, the cholinergic excess or the neuromuscular excitation can provoke respiratory failure. Sometimes, death is due to an anaphylactic reaction to the envenoming. The purpose of this literature review is to evaluate the autopsy findings in scorpion sting-related deaths in order to better understand the pathophysiological mechanisms underlying them, thus helping pathologists in defining the correct diagnosis.

## 1. Introduction

Scorpion sting is a public health issue in several countries, particularly in America, the Middle East, India and Africa [1,2,3,4,5,6,7,8,9,10]. Scorpionism epidemiology in the world is poorly known [11], even if it has been estimated that the annual global incidence is about 1.5 million envenomings, resulting in 2600 deaths [12]. The published studies suggest a higher prevalence and mortality among children [13,14,15,16]. Scorpions are classified in the phylum Arthropoda; their body is lobster-like in shape, with seven sets of paired appendages: the chelicerae, the pedipalps (claws or pincers), four sets of legs and the pectines (comb-like structures on the ventral surface). The segmented tail curves up dorsally, ending in the terminal bulbous segment called the telson, which contains paired venom glands and the aculeus (stinger) [17]. Their origin is dated to approximately 450 million years ago, since when they divided into 19 recognized families and more than 2200 species [18]. It is estimated that about 50 species from the families of Buthidae, which includes the genera *Leiurus* in the Near and Middle East, *Androctonus* and *Buthus* in North Africa, *Tityus* in South America, *Centruroides* in North and Central America, *Mesobuthus* in Asia and *Parabuthus* in Southern Africa. Hemiscorpiidae and Scorpionidae are harmful to humans [18,19]. Scorpion venom is a complex structure composed of neurotoxic proteins, salts, acidic proteins and organic compounds, thereby having neurologic, cardiovascular, hematologic and renal side effects, in addition to local effects such as redness, pain, burning and swelling [1]. However, the effect of the scorpion sting is highly dependent on the species, due to the different targets of the venom [20]. Particularly, while species like *Centruroides* and *Parabuthus* cause neuromuscular issues, *Buthus*, *Mesobuthus* and *Androctonus* exhibit life-threatening cardiovascular effects [21]. Death may be due to the toxic action of the envenoming/poison, but also to an anaphylactic reaction to it [22,23]. Thus, if an autopsy is performed for a suspected death due to a scorpion sting, it can be challenging for the pathologist to get the correct diagnosis. The purpose of this literature review is to evaluate the autopsy findings in scorpion sting-related deaths in order to better evaluate the post-mortem evidence in those cases.

## 2. Materials and Method

The present review of the literature was carried out according to the PRISMA statement [24].

### 2.1. Literature Search

An electronic search of SCOPUS, PubMed and Web of Sciences databases was performed to recognize the relevant research available until 7 August 2020, in order to examine the autopsy findings of fatal scorpion stings. “Fatal” AND “Scorpion” AND “Sting” were used as search terms. The search was further extended by a snowball search and hand searching by using Google Scholar.

### 2.2. Inclusion and Exclusion Criteria

The following inclusion criterion was adopted: (1) papers reporting autopsy cases of a fatal scorpion sting with a description of the findings. The following exclusion criteria were applied: (1) animal studies; (2) scientific article not published in English or French language; and (3) full text not available. For duplicate studies, only the article with more detailed information was included.

### 2.3. Data Extraction

Three researchers (Alessandro Feola, Marco Alfonso Perrone, Amalia Piscopo) independently examined the selected papers. The title, abstract and full text of each potentially pertinent study were reviewed. Any divergence on the eligibility of the studies was determined throughout debate or by consulting an additional reviewer (Giulio Di Mizio). The following information was extracted from all qualified papers: authors, year of publication, country, victim’s age and sex, time interval of sting–death, site of the sting and autopsy features.

## 3. Results

### 3.1. Characteristics of Eligible Studies

**Figure 1:**
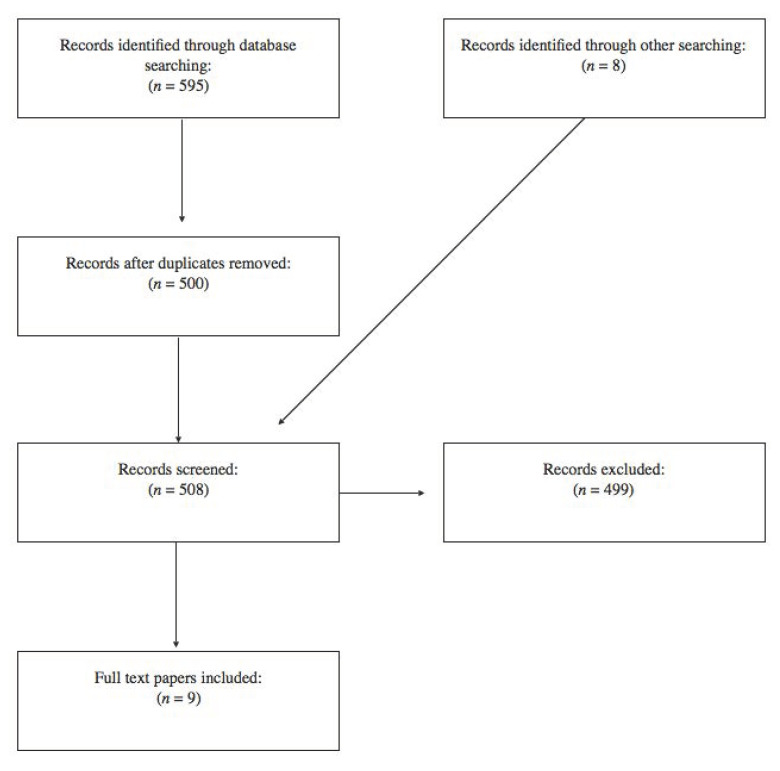
Flowchart depicting the choice of studies.

After a search for scientific literature by the reviewers, a total of 508 documents were screened by the analysis of the title and abstract. In conclusion, nine papers were included in the review. A flowchart depicting the choice of papers is described in Figure 1. A summary of the details of the included research papers is reported in Table 1.

### 3.2. Epidemiological Findings

The papers we reviewed reported cases from Brazil (6), India (5) and Turkey (1). The mean victim’s age was 13.25 years old, and in 58.33% it was <10 years old and M/F was 2:1. The site of the bite was represented in three cases by the upper extremities, in two cases by the trunk and in four cases by the lower extremities. With regard to the time interval between the sting and death, in five cases it was more than 24 h and in four cases less. In these latter cases, the time interval was >12 h.

### 3.3. Autopsy Findings

As known, the external examination findings were not specific and challenging to identify; usually, they are represented by a small puncture mark inside a discolored area. Internal autopsy findings are mainly represented by diffuse congestion and edema. In all the examined cases, lungs showed congestion and alveolar edema with the histological features of ARDS (hyaline membranes, atelectasis, infiltrate of PMNs common in the early stage of ARDS). In four cases, the cardiac findings were represented by PMNs or lymphocyte infiltrate around the papillary muscle and myocytolysis.

## 4. Discussion

**Figure 2:**
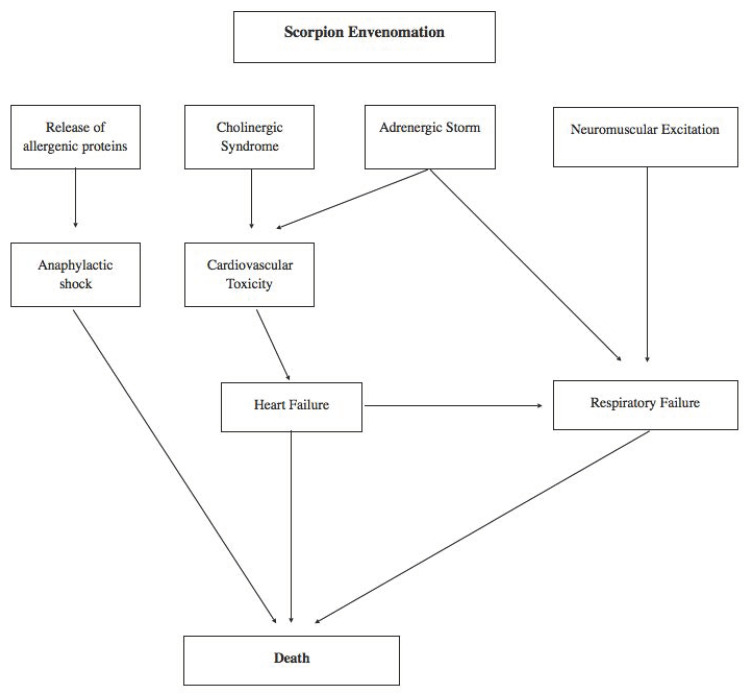
Physiopathological mechanisms leading to scorpion envenomation-related death [34,44].

Most scorpion stings cause localized pain, whereas only an estimated 10% of stings, even from the most dangerous scorpions, result in severe systemic envenomation. Edema, erythema, paresthesia, muscle fasciculations and numbness may occur at the site of the sting [34]. In 2011, Khattabi et al. proposed a three-classes classification of the clinical consequences of scorpion stings [35]. The I class includes local manifestations such as bullous eruption, burning sensation, ecchymosis, erythema, hyperesthesia, itching, necrosis, paresthesia, pain, purpura/petechia, swelling and tingling [35]. The II class includes minor manifestations (non-life-threatening) such as abdominal distension, agitation, anisocoria, arthralgia, ataxia, confusion, convulsion, diarrhea, dry mouth, dystonia, encephalopathy, fasciculation, gastrointestinal hemorrhage, hematuria, headache, hypertension, hyperthermia, hypothermia, lacrimation, local muscular cramps, miosis, mydriasis, myoclonia, nausea, nystagmus, odinophagia, pallor, pancreatitis, general paresthesia, priapism, prostration, ptosis, rhinorrhea, salivation, somnolence, stridor, sweating, tachycardia, thirst, urinary retention, vomiting and wheezing [35]. Finally, the III class includes the most severe and life-threatening scenarios, limited to those patients presenting at least one of the following criteria: (1) cardiogenic failure (hypotension, ventricular arrhythmia, bradycardia and cardiovascular collapse); (2) respiratory failure (cyanosis, dyspnea and pulmonary edema) or/and (3) neurological failure (GCS ≤ 6 in absence of sedation, paralysis) [35]. As reported in most of the cases analyzed in our review, pulmonary edema is one of the key elements in the case of a fatal scorpion sting [25,26,27,28,29,30,31,32,33]. The origin of this finding is challenging [36], but it would be helpful to understand the pathophysiology of fatal scorpion stings. Acute cardiogenic pulmonary edema is one of the manifestations of acute heart failure. It is characterized by ventricular dysfunction, reduced cardiac output and hypoperfusion, increased pulmonary capillary pressure and therefore congestion. The venom can cause myocardial damage by several pathogenetic mechanisms. Myocardial infarction by coronary spasm may occur after the scorpion sting. Indeed, the release of vasoactive, inflammatory and thrombogenic peptides and amine constituents (histamine, bradykinin, serotonin, thromboxane, leukotrienes) can induce coronary artery vasospasm and facilitate platelet aggregation as well as thrombosis [37]. Agrawal et al. showed a case of a 14-year-old boy with myocardial infarction due to vasospasm after a scorpion sting [37]. The venom can also have a direct cardiotoxic effect causing toxic myocarditis and adrenergic myocarditis by releasing adrenaline and noradrenaline, thus increasing the myocardial oxygen demand, resulting in cardiac dysfunction [38]. Bahloul et al. examined the histopathology of two fatal myocarditis cases caused by a scorpion sting, revealing a mixed picture of toxic myocarditis and coagulative myocytolysis, similar to catecholamine-induced cardiomyopathy [39]. Valdivia et al. reported a series of 32 children with scorpion bites who developed cardiac complications. Among these, 50% exhibited myocarditis, 12.5% had subclinical disease and 63% had observed electrocardiographic changes [40]. Another cause of death is due to the release of allergenic proteins that provoke anaphylactic shock leading to hypotension with vasodilation and decrease in the intravascular volume with reduced myocardial perfusion and consequent ischemia and heart failure [41]. Scorpion venom inhibits angiotensin converting enzyme (ACE), resulting in accumulation of bradykinin, which is implicated in the development of pulmonary edema [41]. Noncardiogenic pulmonary edema is a disease process that results in acute hypoxia secondary to a rapid deterioration in respiratory status [42]. The disease process has multiple etiologies, all of which require prompt recognition and intervention. Increased capillary permeability and changes in pressure gradients within the pulmonary capillaries and vasculature are mechanisms for which noncardiogenic pulmonary edema occurs [42]. Acute pulmonary edema corresponds to the brutal flooding of the pulmonary alveoli and therefore to a histological picture characterized by alveolar wall thickening, dilated capillaries and interstitial edema and transudation in the alveolar lumen (granular and pale eosinophilic) [43] as reported in the cases included in our review [25,26,27,28,29,30,31,32,33]. With regard to the physiopathology, it is known that among the many constituents of scorpion venom, alpha toxins produce a significant portion of human toxicity by binding to sodium channels in cell membranes and inhibiting inactivation of the action potential [34]. This action, along with synergistic effects by other venom components, causes prolonged depolarization and excessive release of acetylcholine from parasympathetic ganglia and epinephrine and norepinephrine from sympathetic ganglia and adrenal glands. This excessive release of neurotransmitters provokes an autonomic storm consisting of cardiovascular (tachycardia, peripheral vasoconstriction, hypertension, diaphoresis), metabolic (hyperthermia, hyperglycemia), urogenital (bladder dilatation, urinary retention, ejaculation in males), respiratory (bronchial dilation, tachypnea) and neuromuscular (mydriasis, tremor, agitation, convulsions) complications. More rarely, delayed or masked by the adrenergic storm, a cholinergic (or muscarinic) syndrome can occur involving the parasympathetic nervous system and resulting in a hypersecretion syndrome (salivation, sweating, vomiting, urinary incontinence, bronchial hypersecretion and diarrhea), abdominal pain, miosis, bronchospasm, bradycardia with hypotension and, in the male, priapism [44]. Some of the symptoms could be reinforced and exacerbated by the release of inflammatory substances or vasodilators (kinins, prostaglandins) (Figure 2) [44].

The cardiotoxicity mechanism with acute pulmonary edema of a scorpion sting is still under debate. Likely, it reflects a complex interplay of multiple venom effects, such as catecholamine-mediated coronary, cardiac microcirculatory and systemic vasoconstriction, catecholamine-induced tachycardia, arrhythmias and myocarditis and depression of myocardial contractility caused by direct effects of the scorpion venom and the pro-inflammatory response to envenomation [34,43,45,46,47]. In four of the cases reviewed, a PMNs or lymphocyte infiltrate around the papillary muscle and myocytolysis is described [27,30,31,33]. The diagnosis of scorpion envenomation is based on the evidence of a scorpion sting (by history or recovery of the scorpion) and clinical findings of scorpion envenomation or on suggestive findings in a patient presenting in a region where scorpions are indigenous [48]. Several conditions should be considered in the differential diagnosis, such as: snake bites, spider envenomation, poisoning caused by cholinesterase inhibitors, neuroleptic drug overdose, Guillain–Barré syndrome, tetanus, poliomyelitis, botulism, myasthenia gravis, encephalitis, meningitis, subdural hematoma and diphtheria [49]. The management of scorpion sting is based on the severity of the symptoms [44]. As first aid measures, the National Institute for Occupational Safety and Health (NIOSH) of the U.S.A. suggest to contact a qualified health care provider or poison control center for advice and medical instructions; to apply ice directly to the sting site without submerging the affected area in ice water); to remain relaxed and calm without taking sedatives; and, when possible, to capture the scorpion for identification [50]. In patients with localized and remote pain or paresthesia, treatment consists of pain and wound management [34,44], while in patients with signs of systemic toxicity, the antivenom, Prazosin and supportive care are necessary [34,44].

## 5. Conclusions

Autopsy findings in the case of a fatal scorpion sting are not well-described in the literature and only few case reports or case series are available. Although there is a limited number of published papers, the frequent finding of pulmonary edema and ARDS suggests an impairment of the respiratory function that can be secondary, or not, to the cardiac one. It is also important, in the context of forensic pathology, to report and investigate—with histological and immunohistochemical examinations—this type of death in order to better understand the pathophysiological mechanisms underlying them.

## Figures and Tables

**Table 1:** Summary of the included papers. AG: adrenal glands; ARDS: acute respiratory distress syndrome; B: brain; H: heart; I: intestine; K: kidneys; L: lungs; LA: left atrium; Li: liver; n.a.: not available; P: pancreas; PMNs: polymorphonuclear leukocytes; RV: right ventricle; S: spleen; St: stomach; T: thymus; ↑: increase.

Reference	Country	Scorpion	Age/Sex of the Victim	Time Interval Sting–Death	Site of the Sting	Autopsy Findings
Amaral CSF et al., 1993 [25]	Brazil	Tityus Serrulatus	3/M	57 h	n.a.	H: dilatated RV. Diffuse engorgement of the myocardial vessels and mild interstitial edema L: congested, edematous and stiffened, showing scattered areas of subpleural emphysema, septal thickening, diffuse alveolar edema with prominent hyaline membrane, mononuclear cell infiltration and areas of alveolar collapse
Amaral CSF et al., 1994 [26]	Brazil	Tityus Serrulatus	16/M	32 h	Left Hand	Sampled just the right lung congestion of the alveolar capillaries, widening of the interstitial spaces, focal areas of intra-alveolar edema with marked PMNs infiltration and prominent hyaline membranes lining small bronchioles and alveolar epithelium surface
Cupo P et al., 1994 [27]	Brazil	Tityus Serrulatus	5/F	17 h	Right Supraclavicular Region	B: edema, H: dilatated, pale aspect of the pectineal muscles. Interstitial edema with a moderate inflammatory infiltrate consisting of neutrophilic and some eosinophilic cells. L: ↑volume, diffuse alveolar edema and hemorrhage and polymorphonuclear infiltrate P, Li, I: unremarkable
Cupo P et al., 1994 [27]	Brazil	Yellow Scorpion	4/M	13 h	Sternal region	B: edema. H: enlarged RV L: ↑volume, ↑ consistency, proteinaceous and amorphous material and most intermingled with confluent erythrocytes in the alveoli P, Li, AG: unremarkable
Cupo P et al., 1994 [27]	Brazil	Tityus Serrulatus	4/M	15 h	Hand	B: edema and congestion H: no macroscopic changes. Focal subepicardial hemorrhage. Myocytolysis and unstructured fibrillar cytoplasmic architecture turning into an amorphous eosinophilic mass. PMNs infiltrate around the papillary muscles. L: ↑volume, intense alveolar edema and septal congestion P, Li, I: unremarkable
Das S et al., 2013 [28]	India	n.a.	16/F	n.a. but >24 h	Left Foot. An area (2 × 1 cm) of the skin thickened, indurated and pigmented. Normal subcutaneous. Evidence of hyperkeratosis	Blood stained secretions in the lumen of the larynx and trachea L: heavy, congestion K: congestion Li, H: unremarkable
Das S et al., 2013 [28]	India	n.a.	38/F	n.a.	Right Foot Area of thickening and pigmentation (4 × 3 cm) with hemorrhagic marks. Hemorrhagic aspect of the subcutaneous.	L: congestion with multiple petechial hemorrhages and edema H: edema between the myocytes K: congestion P: unremarkable
Kaya K et al., 2018 [29]	Turkey	n.a.	5/M	n.a. but >14 h	n.a.	L: edema, atelectasis, hyaline membranes and hemosiderin-loaded macrophages (ARDS); Li, K, S, H, B, T: congestion
Kumar L et al., 2012 [30]	India	n.a.	8/M	48 h	Right Foot. Small abrasions over the foot, and no edema or redness.	H: dilatated chambers. Pale in aspect. Lymphocyte infiltrate around the papillary muscles. Myocytolysis (vacuolar degeneration), wavy fibers and focal hyalinization L: liver-like consistency and congestion. Alveoli were filled with homogeneous eosinophilic proteinaceous material P: parenchymal necrosis accompanied by interstitial hemorrhage K: congestion
Mahamuni NM et al., 2016 [31]	India	n.a.	4/M	<24 h	Left Foot. Thickening and pigmentation of the skin (1 × 1cm) at the base of the left little toe.	B, L: petechial hemorrhages, edema and congestion H:LA mononuclear cell infiltration in cardiomyocytes with myocytolysis K: necrosis of tubular epithelium Congestion of all other organs
Melo IMLA et al., 2019 [32]	Brazil	Jaguajir Rochae	44/M	n.a.	n.a.	glottal and lung edema
Patil A et al., 2017 [33]	India	n.a.	12/F	40 h	Left Hand. A dark-brown discoloration and swelling area (1.5 × 1 cm) with a punctured wound mark (0.2 × 0.2 cm) inside thick laminated keratin with unremarkable epidermis and upper dermal congestion	diffuse congestion St, K: congestion and hemorrhagic spots. L: edema, hemosiderin-laden macrophages with focal destruction of alveoli H: edema and pericardial infiltration of lymphocytes. Li: unremarkable
